# Identification of an m6A-Related Long Noncoding RNA Risk Model for Predicting Prognosis and Directing Treatments in Patients With Colon Adenocarcinoma

**DOI:** 10.3389/fcell.2022.910749

**Published:** 2022-07-13

**Authors:** Wanying Liao, Junyu Long, Yiran Li, Fucun Xie, Ziyu Xun, Yanyu Wang, Xu Yang, Yunchao Wang, Kang Zhou, Xinting Sang, Haitao Zhao

**Affiliations:** ^1^ Department of Liver Surgery, State Key Laboratory of Complex Severe and Rare Diseases, Peking Union Medical College Hospital, Chinese Academy of Medical Sciences and Peking Union Medical College, Beijing, China; ^2^ Radiology Department, Peking Union Medical College Hospital, Chinese Academy of Medical Sciences, Beijing, China

**Keywords:** colon adenocarcinoma, N6-methyladenosine, long noncoding RNA, TCGA, prognostic signature

## Abstract

N6-methyladenosine (m6A) and lncRNAs have been implicated in the development of colon cancer, including tumorigenesis, migration, and invasion. However, the specific effect of m6A regulators on lncRNAs is not clear, and m6A-related lncRNAs may be new prognostic biomarkers and may help direct treatment and medication. We identified 29 prognostic m6A-related lncRNAs and constructed a risk model using 12 lncRNAs. The model was an independent prognostic factor and could accurately predict the prognosis. A stable and robust nomogram that combined the model and pathologic stage was constructed. A total of 2,424 differentially expressed genes (DEGs) were identified based on the model. Functional analysis of the DEGs showed that they were associated with tumor progression, helping investigate the underlying biological functions and signaling pathways of the risk model. In addition, the low-risk group based on the risk model had more sensitivity to afatinib, metformin, and GW.441756, and patients with low risk would more likely respond to immunotherapy. Moreover, patients with higher risk were more sensitive to olaparib, bexarotene, and doxorubicin.

## Background

The incidence of colorectal cancer (CRC) is 10.0%, ranking third, and the mortality is 9.4%, ranking second among all types of cancers worldwide according to the newest published cancer statistics (Sung et al., 2021). Colon adenocarcinoma (COAD) is the most frequent subtype (>90%) of CRC (Keum and Giovannucci, 2019). Treatment options for COAD include colectomy, chemotherapy, radiation therapy, and immunotherapy-targeted therapy (Dienstmann et al., 2015). Due to widespread colonoscopy testing and improved therapeutic strategies, the incidence and mortality have been reduced (Siegel et al., 2020). However, the recurrence and metastasis rates within 5 years of treatment of COAD are as high as 30%–50%, and nearly all patients develop drug resistance (Hu et al., 2016). Therefore, reliable molecular biomarkers are urgently needed to provide therapeutic and prognostic information.

N6-methyladenosine (m6A) is a reversible process involving methylation at position N6 of adenosine. It is the most prevalent internal modification in mRNA and long noncoding RNA (lncRNA) in eukaryotes (Zaccara et al., 2019). m6A modification involves methyltransferases (writers), signal transducers (readers), and demethylases (erasers), which are important for controlling many cellular and biological processes (Zaccara et al., 2019). The regulators of m6A have been recently implicated in cancer development, including proliferation, migration, and invasion (Liu et al., 2018). For example, METTL14 is reported to suppress the tumorigenicity and migration of CRC by negatively regulating the lncRNA XIST (Yang et al., 2020). METTL3 enhances CRC progression by stabilizing HK2 and SLC2A1 (GLUT1) and activating the glycolytic pathway (Shen et al., 2020). YTHDF3 downregulates the m6A-modified lncRNA GAS5, which disrupts the interaction between the lncRNA GAS5, leading to CRC progression (Ni et al., 2019). Moreover, lncRNAs were associated with the development of colon cancer, such as regulating cell growth, cell proliferation, cell migratory and invasive abilities, cancer stem cells, and drug resistance (Lan et al., 2018; Chen and Shen, 2020). However, the specific effect of m6A regulators on lncRNAs is not clear, and m6A-related lncRNAs may be new prognostic biomarkers and help find new therapeutic drugs.

In this study, we obtained the expression data of 23 m6A genes and 14,086 lncRNAs from The Cancer Genome Atlas (TCGA) dataset. Then, lncRNAs related to m6A were screened out through Spearman’s correlation analysis. Prognostic m6A-related lncRNAs were screened and used to establish a prognostic signature. A nomogram combining the model and pathologic stage was established as a quantitative scale that could be easily used in the clinic to predict the prognosis of COAD patients. Moreover, the model was used to discover potential chemotherapeutic drugs and predict immunotherapeutic effects.

## Materials and Methods

### Data and Patients

First, the sequencing data and clinical information (age, sex, pathologic stage, T stage, M stage, N stage, grade, and survival time and state) of COAD patient samples were downloaded from the TCGA database (March 2021, https://cancergenome.nih.gov/), including 398 tumor tissues and 39 adjacent normal colon tissues. A total of 379 tumor samples with complete survival information (survival time and state) were randomly grouped into a training dataset and test dataset and used in subsequent analyses. All the data we used were publicly available and had open access, so there was no need for approval from the local ethics committee. The publication guidelines of the TCGA were followed.

### Identification of m6A-Related Long Noncoding RNAs

According to the previously published literature, we identified 23 m6A genes, including eight methyltransferases (METTL3, METTL14, METTL16, WTAP, VIRMA, ZC3H13, RBM15, and RBM15B), 13 signal transducers (YTHDC1, YTHDC2, YTHDF1, YTHDF2, YTHDF3, HNRNPC, FMR1, LRPPRC, HNRNPA2B1, IGFBP1, IGFBP2, IGFBP3, and RBMX) and two demethylases (FTO and ALKBH5). (Arguello et al., 2017; Liu et al., 2017; He et al., 2019). The expression levels of these 23 genes and all 14,086 lncRNAs were extracted from the TCGA database. As the expression data did not fit a normal distribution, Spearman’s correlation analysis was used to screen out m6A-related lncRNAs (absolute correlation coefficient > 0.4 and *p* value < 0.001).

### Construction and Validation of the m6A-Related Long Noncoding RNA Prognostic Signature

Univariate Cox proportional hazards regression (PHR) analysis was implemented to identify prognostic m6A-related lncRNAs according to the survival information of the COAD samples. A *p* value < 0.05 was the threshold indicating that a lncRNA was associated with the prognosis. Next, prognosis-associated lncRNAs were applied to construct an m6A-related lncRNA prognostic signature (m6A-LPS) using samples from the training dataset *via* least absolute shrinkage and selection operator (LASSO) Cox regression analysis. LASSO Cox analysis identified lncRNAs mostly correlated with overall survival with the minimum criteria, and 10-fold cross-validation was performed to prevent overfitting. The constructed formula of the risk model was as follows:

$$\text{Risk score}=\overset{\text{n}}{\underset{\text{i}=1}{\sum }}{\text{Coef}}_{\text{i}}{\text{∗}\mathrm{Exp}}_{\text{i}}.$$

In this formula, Coefi represents the regression coefficient, and Expi represents the expression level of each lncRNA included in the model. Then, we applied the formula to obtain the risk scores for patients in the test set. The median risk score of the training set was identified, based on which all the 379 patients were divided into high- and low-risk groups. Thereafter, the results of Kaplan–Meier survival analysis for COAD patients in the two groups and receiver operating characteristic (ROC) curve analysis of the 1-, 2-, and 3-year survival rates were used as indicators for evaluating the predictive ability and accuracy of m6A-LPS.

### Independence of m6A-LPS in Predicting Prognosis

To evaluate the independence of m6A-LPS in predicting the overall survival (OS), univariate and multivariate Cox regression analyses were implemented. Age, sex, and pathologic stage were all considered. Two-sided *p* values, hazard ratios (HRs), and 95% confidence intervals (CIs) were calculated.

### Establishment and Validation of the Nomogram

To establish a quantitative scale that could be easily used in the clinic to predict the prognosis, a nomogram was established. It combined two independent risk factors, m6A-LPS and pathologic stage. Calibration plots were visualized using the R package “rms”. To validate the nomogram in terms of its predictive accuracy, ROC analysis was performed.

### Functional Analysis

With sex and age applied as confounding factors, we identified the differentially expressed genes (DEGs) between the two groups *via* the R package “limma”. The adjusted *p* value was calculated using the Benjamini–Hochberg method. Next, DEGs with an absolute log2-fold change (FC) > 1 and an adjusted *p* value of <0.05 were used in Gene Ontology (GO) and Kyoto Encyclopedia of Genes and Genomes (KEGG) pathway enrichment analyses. This process utilized the R package “clusterProfiler” to annotate the GO and KEGG functions, with an adjusted *p* value <0.05 and an overlapping gene number ≥3 as the thresholds for indicating that the DEGs were significantly enriched in a functional term.

### Exploration of Potential Chemotherapeutic Drugs

To explore the potential chemotherapeutic drugs for COAD treatment, we calculated the half-maximal inhibitory concentration (IC_50_) of compounds obtainable in the Genomics of Drug Sensitivity in Cancer (GDSC) website for all the COAD samples using the R package “pRRophetic”. We compared the IC_50_ values between the low- and high-risk groups and explored the correlation between the IC_50_ value and each prognostic m6A-related lncRNA *via* linear regression analysis.

### Prediction of Immunotherapeutic Treatment

The expression levels of 47 common immune checkpoint genes in patients with low and high risk were extracted and compared, including the two commonly used therapies, which targets programmed cell death protein 1 (PD-1) and cytotoxic T-lymphocyte antigen 4 (CTLA-4). To explore the role of m6A-LPS in predicting immune treatment, we used the Tumor Immune Dysfunction and Exclusion (TIDE) algorithm (Jiang et al., 2018) and the immunophenoscore (IPS) from The Cancer Immunome Atlas (https://tcia.at/)

## Results

### Identification of Prognostic m6A-Related Long Noncoding RNAs

**FIGURE 1:**
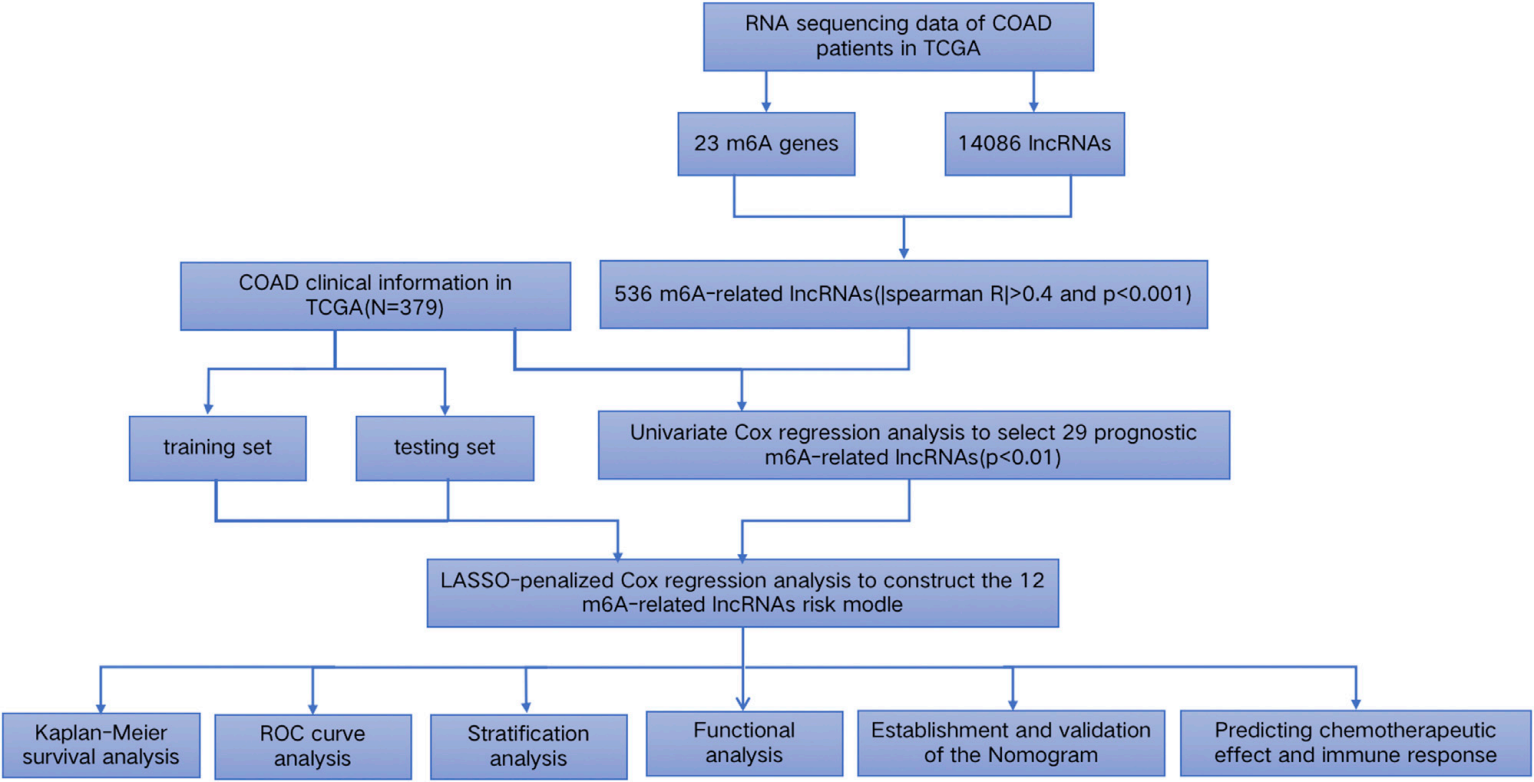
Study workflow. First, the sequencing data and clinical information of COAD patient samples were downloaded from the TCGA database. Second, the expression data of 23 m6A genes and 14,086 lncRNAs were extracted, and 536 lncRNAs related to m6A were screened out *via* Spearman’s correlation analysis. Third, 379 patients with complete survival information were randomly grouped into a training dataset and a test dataset. Fourth, 29 prognostic m6A-related lncRNAs were identified through the univariate Cox PHR analysis according to the survival information of the COAD samples. Then, the 29 lncRNAs were applied to construct the 12 m6A-LPS. Finally, we performed the Kaplan–Meier survival analysis, ROC curve analysis, stratification analysis, and functional analysis, established and validated the nomogram, and predicted the chemotherapeutic effect and immune response of the high- and low-risk groups.

**FIGURE 2:**
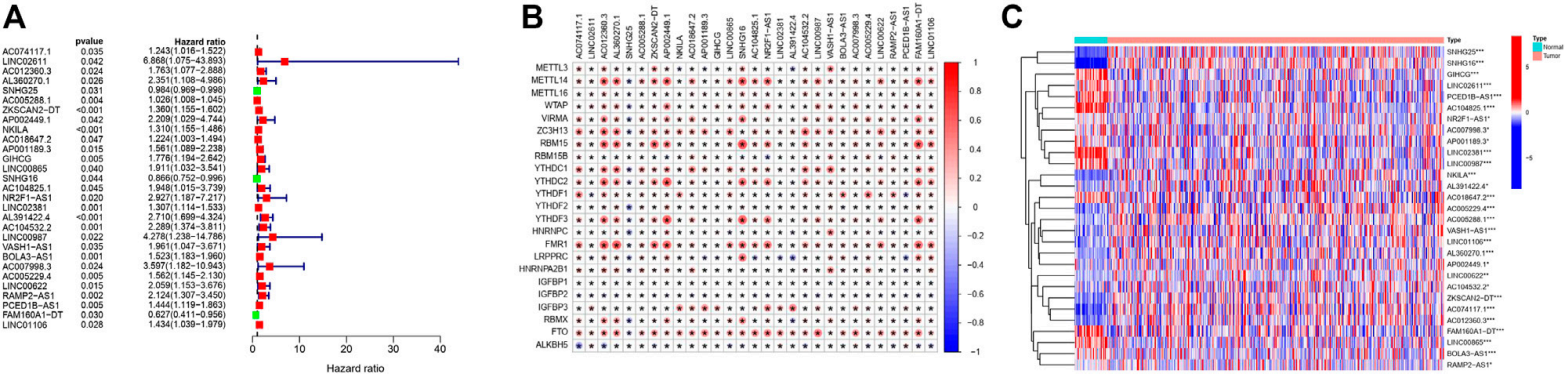
Identification of prognostic m6A-related lncRNAs. **(A)** Forest plot of the results of univariate Cox PHR analysis, including the HR and p values of 29 m6A-related prognostic lncRNAs. **(B)** Heatmap of the correlations between m6A genes and the 29 prognostic m6A-related lncRNAs. The color represents the coefficient. *p* < 0.05 (*). **(C)** Heatmap of the expression levels of the 29 m6A-related prognostic lncRNAs in the COAD samples and adjacent normal samples. *p* < 0.05 (*), *p* < 0.01 (**), and *p* < 0.001 (***).

The whole study design is visualized in Figure 1. A total of 379 samples from COAD patients with the survival state and time information were included and randomly divided into the training and testing sets. Table 1 shows the age and sex characteristics and clinical information of the 379 patients. A total of 536 lncRNAs were identified to be m6A-related (|Spearman R|>0.4 and *p* < 0.001) and 29 were prognosis-related (*p* < 0.05, Figure 2A). Detailed information on the 29 prognosis-related lncRNAs is shown in Table 2, and their correlation with the 23 m6A genes is shown in Figure 2B. All the 29 prognosis-related lncRNAs had markedly different expression levels between the tumor and normal samples (Figure 2C).

**TABLE 1:** Age and sex characteristics and clinical information of COAD patients.

Variables	Type	Total	Test	Train	*p* value
Sex	Female	178 (46.97%)	89 (47.34%)	89 (46.6%)	0.9664
	Male	201 (53.03%)	99 (52.66%)	102 (53.4%)	
Age	≤65	157 (41.42%)	74 (39.36%)	83 (43.46%)	0.481
	>65	222 (58.58%)	114 (60.64%)	108 (56.54%)	
Stage	Stage I–II	213 (56.2%)	109 (57.98%)	104 (54.45%)	0.4301
	Stage III–IV	155 (40.9%)	72 (38.3%)	83 (43.46%)	
	Unknown	11 (2.9%)	7 (3.72%)	4 (2.09%)	
T	T1–2	76 (20.05%)	40 (21.28%)	36 (18.85%)	0.6624
	T3–4	302 (79.68%)	148 (78.72%)	154 (80.63%)	
	Unknown	1 (0.26%)	0 (0%)	1 (0.52%)	
M	M0	281 (74.14%)	138 (73.4%)	143 (74.87%)	0.27
	M1	53 (13.98%)	31 (16.49%)	22 (11.52%)	
	Unknown	45 (11.87%)	19 (10.11%)	26 (13.61%)	
N	N0	227 (59.89%)	119 (63.3%)	108 (56.54%)	0.2163
	N1–2	152 (40.11%)	69 (36.7%)	83 (43.46%)	

**TABLE 2:** Detailed information on the 29 m6A-related lncRNAs.

lncRNA	HR	HR.95L	HR.95H	*p* value
AC074117.1	1.24345294	1.01561087	1.52240909	0.03486142
LINC02611	6.86757852	1.07452082	43.8927136	0.04175956
AC012360.3	1.7632967	1.07656969	2.88807614	0.02425657
AL360270.1	2.35087063	1.10831388	4.98648696	0.02587929
SNHG25	0.98357902	0.96890114	0.99847925	0.03090027
AC005288.1	1.02643952	1.00836653	1.04483643	0.00398665
ZKSCAN2-DT	1.36033059	1.15518945	1.60190116	0.00022449
AP002449.1	2.20910405	1.02866702	4.74414034	0.04210901
NKILA	1.31010273	1.15507238	1.48594078	2.63E-05
AC018647.2	1.22386226	1.00260409	1.49394846	0.04708602
AP001189.3	1.56119807	1.08927432	2.23758091	0.01528334
GIHCG	1.77591479	1.19386171	2.64174093	0.00458995
LINC00865	1.91113998	1.03152243	3.54084013	0.03953199
SNHG16	0.86552743	0.75191477	0.99630672	0.04427255
AC104825.1	1.94847742	1.01531235	3.73930668	0.044893
NR2F1-AS1	2.92661713	1.18673627	7.21734731	0.01971558
LINC02381	1.30688973	1.11404368	1.53311831	0.00101706
AL391422.4	2.71040257	1.69906239	4.3237271	2.86E-05
AC104532.2	2.28854142	1.3742931	3.81099332	0.00146319
LINC00987	4.27817822	1.23787788	14.7856337	0.02160565
VASH1-AS1	1.96093609	1.04741052	3.67121609	0.0353142
BOLA3-AS1	1.52287532	1.18331737	1.95987085	0.00108439
AC007998.3	3.5965658	1.18202913	10.9432883	0.02416328
AC005229.4	1.56170203	1.14517109	2.129737	0.00485673
LINC00622	2.05874218	1.15285605	3.67645152	0.01465646
RAMP2-AS1	2.12372599	1.30748859	3.44952311	0.00234007
PCED1B-AS1	1.44369215	1.11861556	1.86323801	0.00478536
FAM160A1-DT	0.62655444	0.41083672	0.95553891	0.02991807
LINC01106	1.43389894	1.03920176	1.97850529	0.02823102

### Construction and Validation of the m6A-Related Long Noncoding RNA Prognostic Signature

**FIGURE 3:**
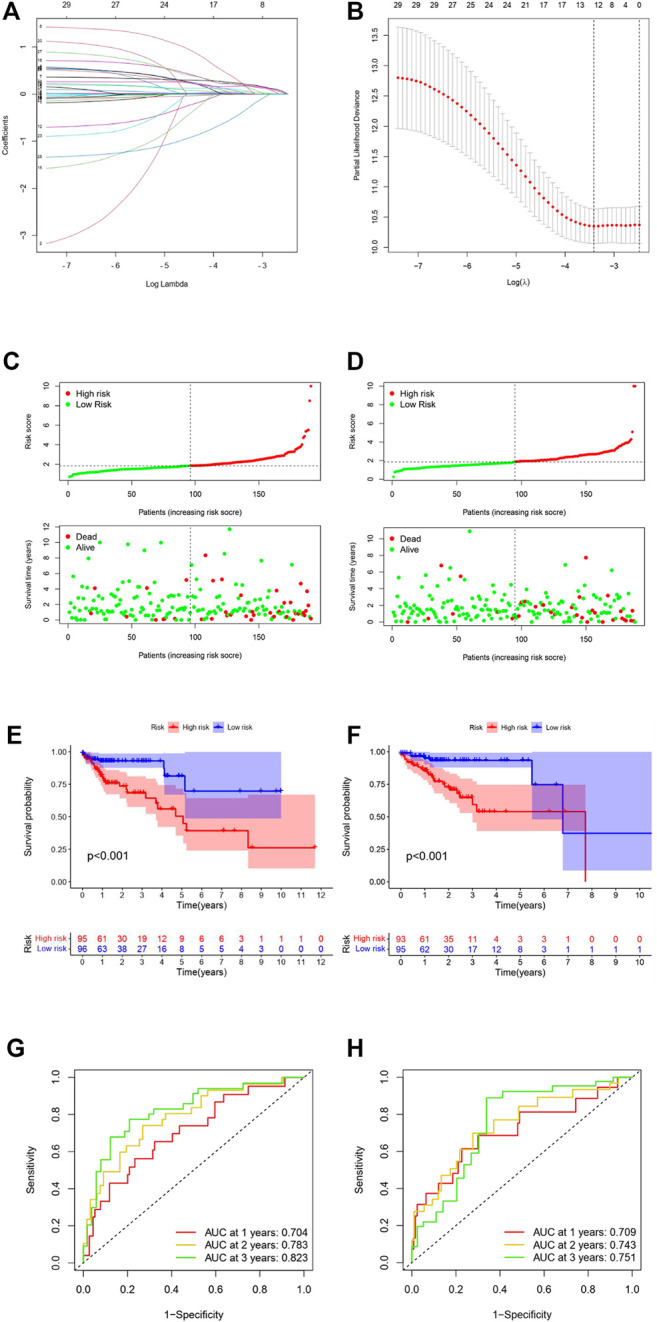
Construction and validation of the risk model. **(A,B)** LASSO Cox analysis identified the best parameters and optimal coefficients. **(C,E,G)** Analysis results of the training set. **(D,F,H)** Analysis results of the testing set. **(C,D)** Risk score distribution of m6A-LPS (panel above) and patient survival status (panel below). The green dots represent surviving patients, and the red dots represent non-surviving patients **(E,F)** Survival analysis revealed that the low-risk group patients had a better prognosis. **(G,H)** ROC curves of m6A-LPS for predicting 1-, 2-, and 3-year survival.

LASSO-penalized Cox regression analysis was used to build a prognostic signature based on the 29 m6A-related prognostic lncRNAs in the training set. An m6A-LPS involving 12 lncRNAs was developed (Figures 3A,B). The relationship between the 12 lncRNAs used in m6A-LPS and their related m6A genes is shown in Supplementary Figure S1. The coefficient of each lncRNA used in the m6A-LPS is shown in Table 3. Then, the patients of the entire set were divided into high- and low-risk groups according to the median risk score (Figures 3C,D). AC005288.1, ZKSCAN2-DT, AP002449.1, NKILA, AC018647.2, AL391422.4, BOLA3-AS1, AC005229.4, PCED1B-AS1, and LINC01106 were the risk factors associated with high risk. In contrast, the other two, SNHG25 and FAM160A1-DT, tended to be favorable factors in COAD patients. Kaplan–Meier survival analysis was performed and indicated that the OS (Figures 3E,F) and progression-free survival (PFS) (Supplementary Figure S2) of patients with high risk were significantly worse. The results of ROC curve analysis of the 1-, 2-, and 3-year survival rates of the training and testing sets are shown in Figure 3G (1-, 2-, and 3-year AUCs = 0.704, 0.783, and 0.823) and Figure 3H (1-, 2-, and 3-year AUCs = 0.709, 0.743, and 0.751), respectively.

**TABLE 3:** Coefficient of each lncRNA.

lncRNA	Coef
SNHG25	−0.0008713
AC005288.1	0.01068947
ZKSCAN2-DT	0.22086036
AP002449.1	0.22613408
NKILA	0.04928804
AC018647.2	0.01532816
AL391422.4	0.1946172
BOLA3-AS1	0.0313515
AC005229.4	0.1751108
PCED1B-AS1	0.1716126
FAM160A1-DT	−0.3875861
LINC01106	0.08115007

### Stratification Analysis and Functional Analysis

**FIGURE 4:**
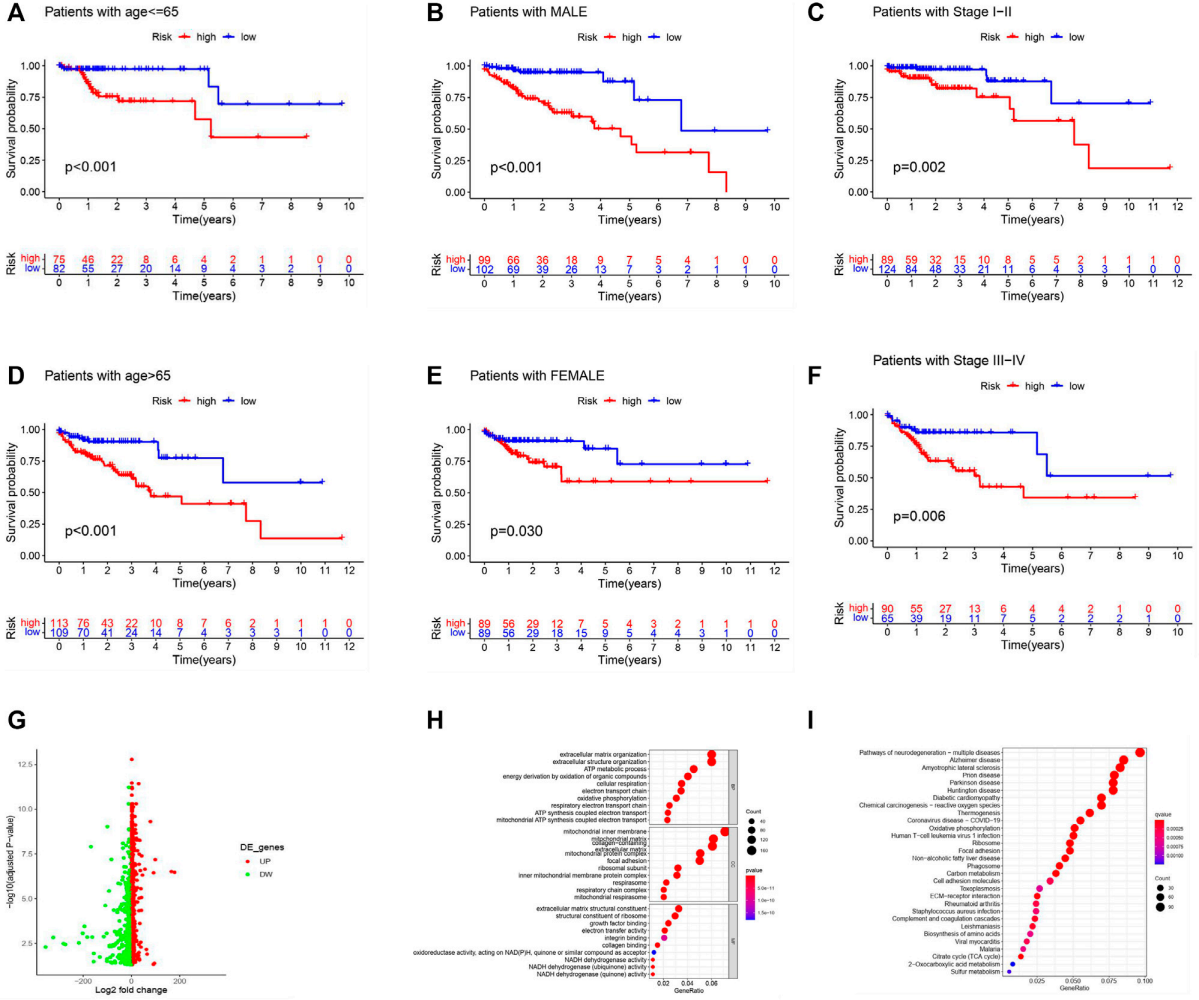
Stratification analysis and functional analysis. **(A–F)** Kaplan–Meier survival analysis showed that patients with high risk always had a worse OS when stratified by age **(A,D)**, sex **(B,F)**, and stage **(C,F)** in the entire dataset. **(G)** Volcano plot showing the DEGs. **(H,I)** GO **(H)** and KEGG pathway **(I)** analyses (*p* < 0.05) of the 2424 DEGs. The size of the circles indicates the gene count, and the color indicates the *p* value.

We investigated the differences in OS of all the COAD patients stratified by the clinicopathological variables between the two groups. Patients with high risk always had a worse OS when stratified by age, sex, and stage (Figures 4A–F). Moreover, to better explore the potential functions of m6A-LPS in COAD, 2,424 DEGs were identified (Figure 4G) and GO functional analysis and KEGG pathway analysis for biological functions were implemented (Figures 4H,I). The DEGs were enriched in some GO terms, including oxidative phosphorylation, focal adhesion, growth factor binding, and positive regulation of cell growth (Supplementary Table S1). They were also enriched in the NADH dehydrogenase activity signaling pathway (Supplementary Table S2). Functional analysis revealed that m6A-LPS is essential in the complex functions of tumors.

### Establishment and Validation of the Nomogram

**FIGURE 5:**
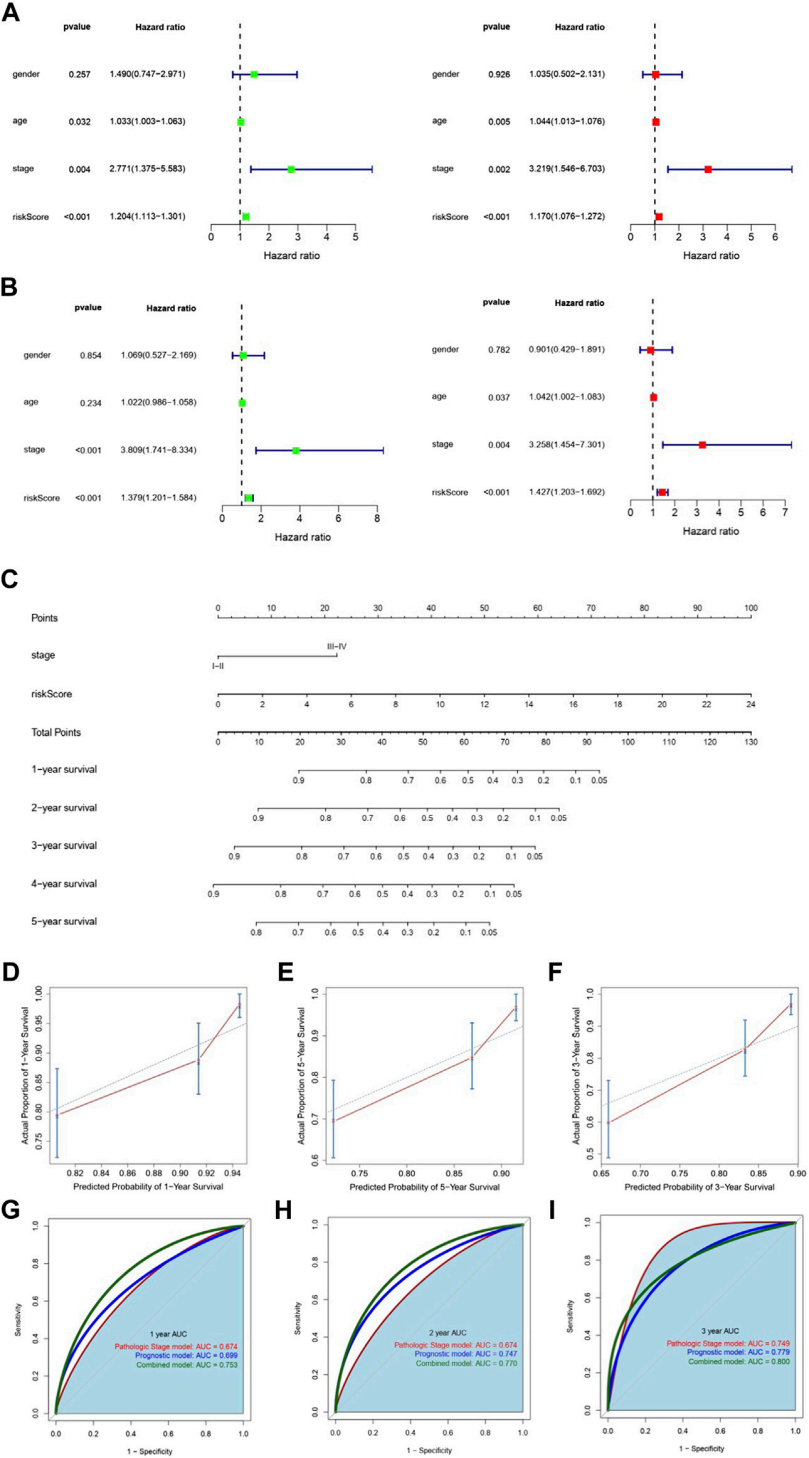
Establishment and validation of the nomogram. **(A,B)** Univariate and multivariate regression analyses showed that m6A-LPS and pathological stage were independent prognostic predictors in both the training set **(A)** and testing set **(B)**. The left and right pictures represent the univariate analysis and multivariate analysis, respectively. **(C)** Nomogram combining m6A-LPS and stage. **(D–F)** Calibration curves for predicting 1-, 2-, and 3-year OS for patients with COAD. **(D–F)** Smooth ROC curves of 1-, 2-, and 3-year OS based on the pathological stage, m6A-LPS, and the nomogram.

Univariate Cox regression and multivariate Cox regression analyses were utilized with the TCGA training and testing sets to detect whether m6A-LPS or clinical characteristics were significantly associated with OS and determine their independence in predicting the prognosis (Figures 5A,B). Then, the pathologic stage and m6A-LPS, two independent prognostic factors, were selected to construct a prognostic nomogram in the entire TCGA cohort (Figure 5C). Calibration plots showed that the observed 1-, 2-, and 3-year OS rates had good concordance with the predicted rates (Figures 5D–F). Then, the area under the curve (AUC) of the 1-, 2-, and 3-year survival rates of the nomogram, risk scores, and pathologic stage was assessed (Figures 5G–I), which revealed that the nomogram had the best predictive ability.

### Exploration of Potential Chemotherapeutic Drugs

The IC_50_ values of compounds available on the GDSC website were calculated to explore potential chemotherapeutic drugs for COAD treatment. Seventy-eight compounds had significantly different IC_50_ values, and most of them had a higher IC_50_ in low-risk patients, including olaparib (AZD.2281), bexarotene, and doxorubici (Supplementary Figures S3A–C), indicating that patients with higher risk were more sensitive to these drugs. In addition, afatinib (BIBW2992), metformin, and GW.441756 were more useful for low-risk patients (Supplementary Figures S3D–F). The correlation coefficients between the IC_50_ values of olaparib, bexarotene, doxorubicin, afatinib, metformin, and GW.441756 and each prognostic m6A-related lncRNA were calculated. Correlation coefficients with a *p* value <0.05 are summarized in Supplementary Table S3.

### Prediction of Immune Response

**FIGURE 6:**
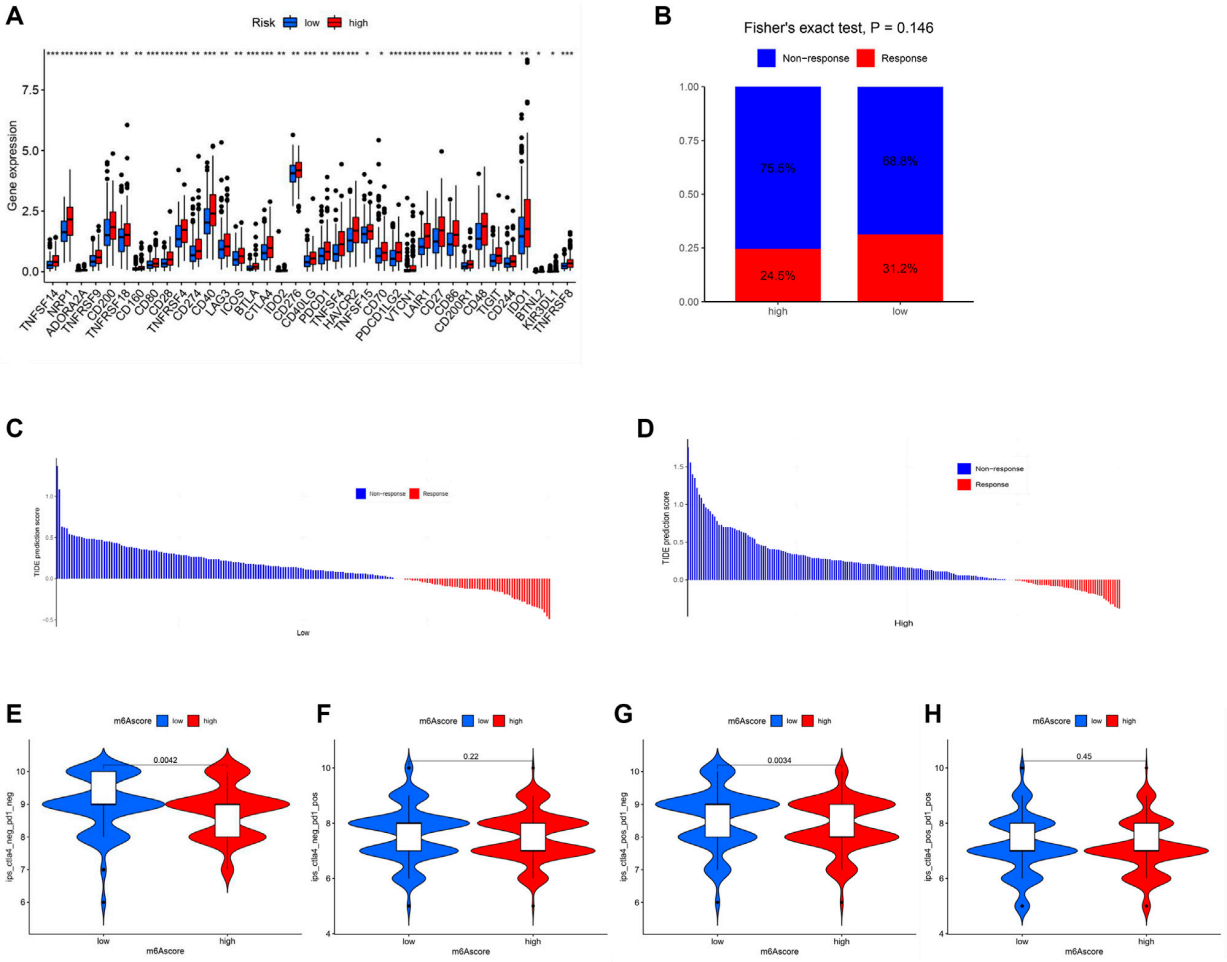
Prediction of immunotherapeutic treatment. **(A)** Expression of 37 immune checkpoint genes was significantly different between high- and low-risk patients. *p* < 0.05 (*), *p* < 0.01 (**), and *p* < 0.001 (***). **(B)** Box plot of the TIDE prediction scores of COAD patients. **(C,D)** Waterfall plot of the TIDE prediction scores of COAD patients. **(E–H)** Immunophenoscore of the high- and low-risk groups from TCIA.

The expression levels of 47 immune checkpoint genes, including PD-1 and CTLA-4, were compared in patients with low and high risk. Thirty-seven genes with significantly different expression levels were identified and visualized (Figure 6A). All the 37 immune checkpoint genes were expressed more actively in the high-risk patients. To explore the role of m6A-LPS in predicting the immune response, the TIDE algorithm (Figures 6B–D) and the IPS from TCIA (Figures 6E–H) were used. The results showed that patients in the low-risk group may have a better response to immunotherapy and had a higher IPS to CTLA4 inhibitor therapy.

## Discussion

m6A regulators were reported to be critical players in cancer tumorigenesis, progression, and metastasis by regulating lncRNAs (Chen et al., 2020; Yi et al., 2020). m6A modification of the lncRNA NEAT1-1 plays a critical role in promoting bone metastasis of prostate cancer (Wen et al., 2020). m6A modification upregulates the expression level of the lncRNA RP11, resulting in the upregulation of Zeb1, which promotes the metastasis of CRC (Wu et al., 2019). METTL3 facilitates hepatocellular carcinoma (HCC) lipogenesis and progression through the m6A modification of LINC00958 (Zuo et al., 2020). The demethylation of the lncRNA NEAT1 caused by ALKBH5, an m6A demethylase, promotes the progression of colon cancer (Guo et al., 2020a). In summary, m6A modification of lncRNAs is critical in the occurrence and progression of tumors, and m6A-related lncRNAs should be considered to discover reliable and robust molecular biomarkers for COAD.

Our study identified 29 prognosis-associated, m6A-related lncRNAs based on data from 379 COAD patients, and 12 of them were used to construct the m6A-LPS. NKILA and BOLA3-AS1 have been identified as unfavorable factors for colon cancer (Xu et al., 2021a; Guo et al., 2021; Chen et al., 2022; Qiu et al., 2022), which is consistent with the findings of this study. PCED1B-AS1 was reported to promote the proliferation of COAD by regulating the miR-633/HOXA9 axis, which was proven by *in vitro* and *in vivo* experiments (Liu et al., 2022). LINC01106 was also reported to drive the growth of CRC and lead to poor prognosis (Guo et al., 2020b). The other eight lncRNAs have not been reported in CRC, and further research and experiments on these lncRNAs might provide new insights.

According to the median risk score, the 379 COAD patients were divided into high- and low-risk groups. Kaplan–Meier survival analysis was performed and indicated that patients in the high-risk group had significantly worse OS. The results of ROC curve analysis of the 1-, 2-, and 3-year survival rates proved the high accuracy of m6A-LPS. Univariate and multivariate Cox regression analyses identified m6A-LPS and pathologic stage as independent indicators in predicting the prognosis. However, the outcomes of COAD patients at the same stage could be very different clinically. We performed the stratification analysis and found that m6A-LPS could perfectly identify the prognosis of patients in the same stage. Moreover, to establish a quantitative scale that could be easily used in the clinic, we constructed a nomogram that combined m6A-LPS and pathologic stage, two independent indicators. Calibration plots and AUC proved that the nomogram was stable and robust and had high accuracy.

To search for the underlying signaling pathways and biological processes of the lncRNAs in the model, 2,424 DEGs were identified and used for functional analysis. The DEGs were enriched in oxidative phosphorylation, focal adhesion, growth factor binding, positive regulation of cell growth, and the NADH dehydrogenase activity signaling pathway. In recent years, oxidative phosphorylation has become a novel target in cancer therapy (Ashton et al., 2018), and combination with targeting oxidative phosphorylation can improve the efficacy of radiotherapy and immunotherapy (Boreel et al., 2021). Focal adhesion plays a key role in tumor invasiveness and metastasis, drug resistance, and radioresistance (Eke and Cordes, 2015). The NADH dehydrogenase complex has been reported to be overexpressed in metastatic mouse colon tumor cells (Marquez et al., 2019). In conclusion, functional analysis provided new insights into the process and mechanism of m6A-lncRNAs in COAD.

In addition, m6A-LPS was used to investigate potential chemotherapeutic drugs and immunotherapeutic targets. The IC_50_ values of chemotherapeutic drugs available on the GDSC website were calculated, which indicated that the low-risk group was more sensitive to almost all the drugs, including olaparib, bexarotene, and doxorubicin, which were reported to be helpful for preventing or treating COAD (Bowen et al., 2021; de Castro e Gloria et al., 2021; Pan et al., 2021). Afatinib, metformin, and GW.441756 are more useful for low-risk patients. Afatinib, a drug mainly used for non–small-cell lung cancer (NSCLC) patients (Park et al., 2016), has a good effect in treating HER2-mutant CRC cell lines. (Kloth et al., 2016). Metformin, an antidiabetic drug, has been proven to be an effective anticancer agent for CRC (Kamarudin et al., 2019). GW441756, a TRKA kinase inhibitor being researched *in vitro*, has been reported to reduce the proliferation and metastasis of pancreatic cancer (Bapat et al., 2016) but has not been studied in COAD. The expression levels of PD-1 and CTLA-4, two immune checkpoint genes, were significantly different between the patients with high and low risk. Low-risk COAD patients were predicted to have a significantly higher IPS to CTLA4 target therapy. Consistently, the prediction results from TIDE showed that patients with low risk would more likely respond to immunotherapeutic therapy, although the difference was not significant.

In conclusion, we constructed a 12 m6A-related lncRNA risk model and a nomogram combining m6A-LPS and pathologic stage, which could help predict the OS and PFS of COAD patients and provide a direction on treatment and medication. In addition, we investigated the underlying biological functions and signaling pathways of m6A-LPS, which provided new insights into the process and mechanism of m6A-lncRNAs in COAD and helped identify new therapeutic targets.

In recent years, with the development of biotechnology and the emergence of public databases, bioinformation technology has played an increasingly important role. There have been several previously established lncRNA-based prognostic models for patients with COAD (Sun et al., 2020; Chen et al., 2021; Ren et al., 2021; Huang et al., 2022). This study was the first to establish an m6A-related lncRNA risk model to predict the prognosis, guide immunotherapy, and discover potential chemotherapeutic drugs. Furthermore, the specific effect of m6A regulators on lncRNAs is not clear, and m6A-related lncRNAs may be new effective prognostic biomarkers and may help direct treatment and medication. Xu et al. (2021b) identified 83 protective and 18 risk m6A-related tissue-elevated lncRNAs across all cancer types. They reported that HCC patients with low expression levels of F11-AS1 and LINC01018 and low-grade glioma and glioblastoma multiforme patients with low expression levels of MIR325HG had a worse prognosis (Xu et al., 2021b). However, Xu et al. (2021b) did not identify the specific m6A-lncRNAs associated with the prognosis of COAD patients. Moreover, this study established a nomogram, which is a quantitative scale that can be easily used in the clinic to predict the prognosis of COAD patients.

The risk model has some limitations. First, the association between lncRNAs and m6A genes was determined by a computational method, lacking experimental evidence. We found valuable published m6A and lncRNA data for normal colon tissues (Liu et al., 2020), but data from the COAD samples may be preferable. Second, further experimental and clinical evidence is needed to explore the molecular mechanisms and verify our prediction results. Nonetheless, in consideration of the limited number of samples, the model needs further validation in other cohorts.

## Data Availability

The datasets presented in this study can be found in online repositories. The names of the repository/repositories and accession number(s) can be found in the article/Supplementary Material.

## Supplementary Material

Supplementary Table S1 Results of Gene Ontology (GO) pathway enrichment analysis.

Supplementary Table S2 Results of Kyoto Encyclopedia of Genes and Genomes (KEGG) pathway enrichment analysis.

Supplementary Table S3 Correlation coefficients between IC_50_ value and each prognostic m6A-related lncRNA. p < 0.05 (*), p < 0.01 (**), and p < 0.001 (***).
