# Supplementary material for: Identification of an m6A-Related Long Noncoding RNA Risk Model for Predicting Prognosis and Directing Treatments in Patients With Colon Adenocarcinoma
Source: Front Cell Dev Biol. 2022 Jul 13;10:910749. doi: 10.3389/fcell.2022.910749 (PMC9326028; doi:10.3389/fcell.2022.910749)
Supplement: Supplementary file 2 [file Table1.DOCX]

**
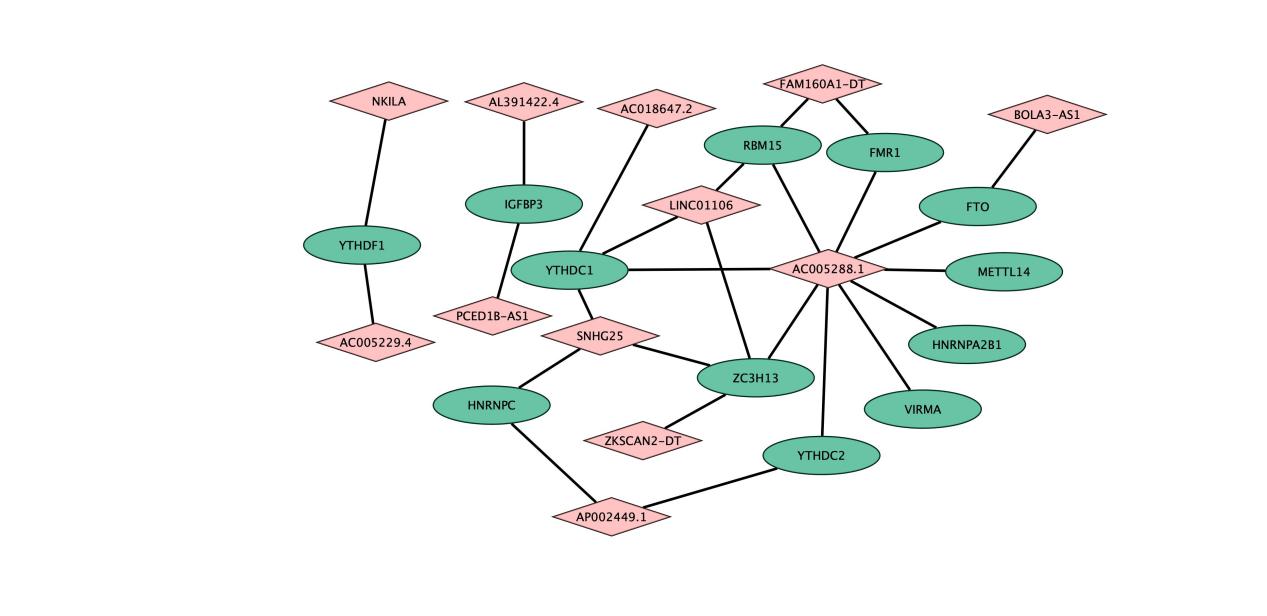
**

**Figure S1.** The Relationship between the 12 m6A-related lncRNAs and related m6A genes. Green ovals represent genes, and pink diamonds represent lncRNAs.


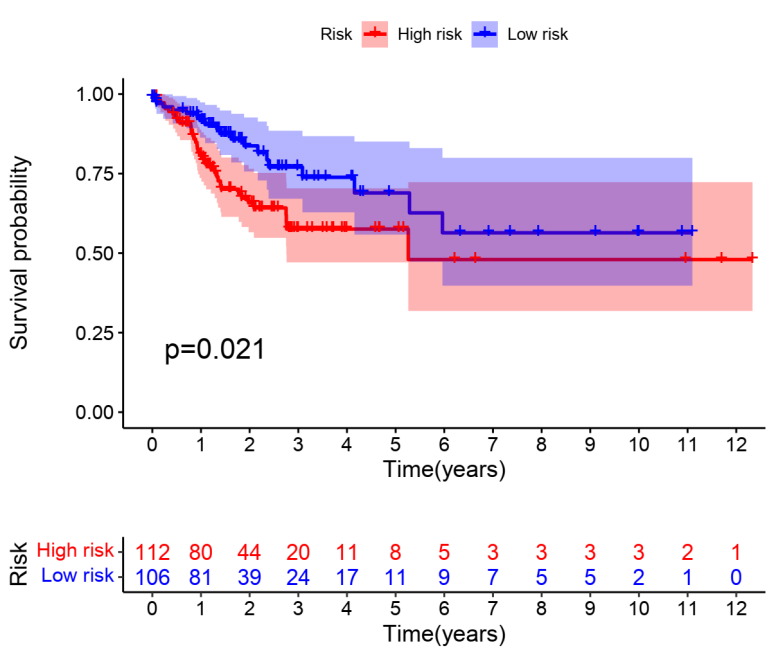


**Figure S2.** Kaplan-Meier survival analysis showed that patients with high risk always had a worse PFS.


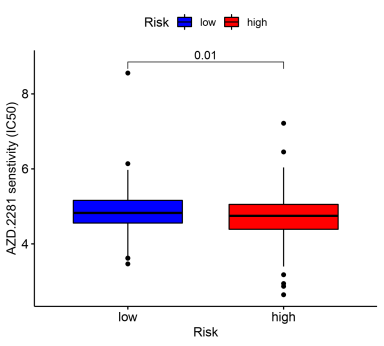

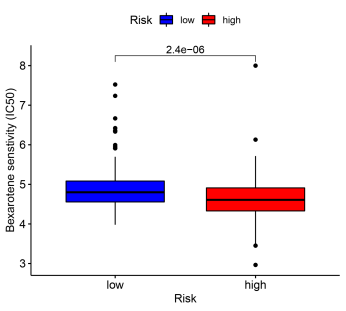

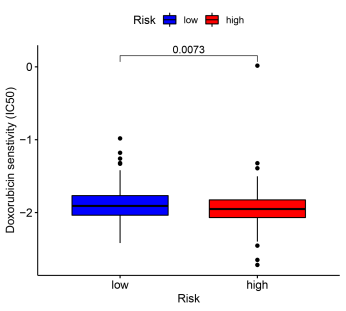


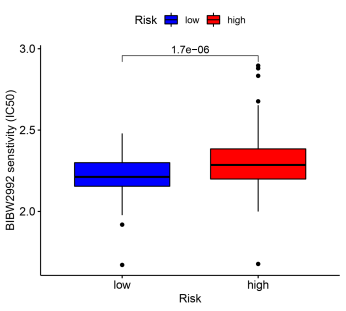

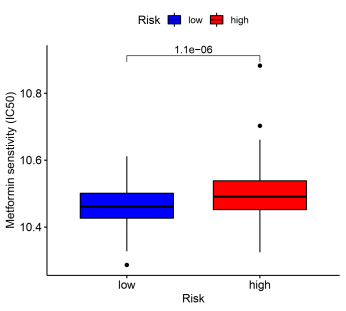

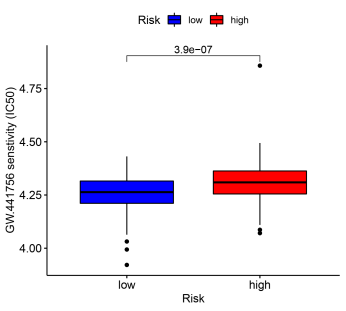


**Figure S3. The IC_50_ of potential chemotherapeutic drugs**
