# Supplementary material for: Identification of an m6A-Related Long Noncoding RNA Risk Model for Predicting Prognosis and Directing Treatments in Patients With Colon Adenocarcinoma
Source: Front Cell Dev Biol. 2022 Jul 13;10:910749. doi: 10.3389/fcell.2022.910749 (PMC9326028; doi:10.3389/fcell.2022.910749)
Supplement: Supplementary file 4 [file Table2.DOCX]

https://www.jianguoyun.com/p/DRH0b5YQwIOdChiovr4EIAA
